# Point-of-care HIV testing best practice for early infant diagnosis: an implementation study

**DOI:** 10.1186/s12889-019-6990-z

**Published:** 2019-06-11

**Authors:** Elizabeth Spooner, Kerusha Govender, Tarylee Reddy, Gita Ramjee, Noxolo Mbadi, Swaran Singh, Anna Coutsoudis

**Affiliations:** 1 Department of Paediatrics and Child Health, School of Clinical Medicine, University of KwaZulu-Natal, Durban, South Africa; 2 Department of Virology, Inkosi Albert Luthuli Central Hospital, National Health Laboratory Service, Durban, KwaZulu-Natal South Africa; 3 South African Medical Research Council, Biostatistics Unit, Durban, South Africa; 4 South African Medical Research Council, HIV Prevention Research Unit, Durban, South Africa; 5 Department of Paediatrics, Addington Hospital, Durban, South Africa

**Keywords:** Early infant diagnosis (EID), PMTCT, Infant HIV, Point-of-care (POC), Maternal viral-load, Alere™q HIV-1/2 detect

## Abstract

**Background:**

With Universal Health Coverage and Integrated People-centred Health Care, streamlined health-systems and respectful care are necessary. South Africa has made great strides in prevention of mother-to-child transmission (PMTCT) but with the great burden of HIV, a minimum of birth and 10-week HIV-PCR testing are required for the estimated 360,000 HIV-exposed infants born annually which presents many challenges including delayed results and loss to follow-up. Point-of-care (POC) HIV testing of infants addresses these challenges well and facilitates initiation of HIV-infected infants rapidly after diagnosis for best clinical outcomes.

**Methods:**

Objectives were to determine accuracy, feasibility and acceptability of POC testing compared to standard-of-care (SOC) central-laboratory testing. HIV-exposed infants for birth PCR testing in hospital (*n* = 323) and follow-up at a primary health care clinic (*n* = 117) in Durban, South Africa were included. A baseline situational-analysis reviewed registers and phoned mothers of HIV-exposed infants prior to the intervention. An effectiveness-implementation study of the Alere™q HIV-1/2 Detect POC test (heel-prick specimen processed in 50 min) was compared with SOC with questionnaires to mothers and staff. Stata 14 was used for analysis.

**Results:**

At baseline 2% of birth HIV tests were missed; only 40% of mothers could be contacted; 17% did not receive birth test result; 19% did not have a 10-week test; 39% had not received the 10-week results. There were 5(1.5%) HIV-infected and 318(98.5%) HIV-negative infants detected in hospital with all clinic babies negative. All positive infants commenced ART before discharge. Ultimately POC and SOC had perfect concordance but for 10 SOC tests researchers actively tracked-down results or repeated tests. Turn around times for SOC tests were on average 8-days (IQR 6-10  days) and for POC testing was 0-days. The POC error-rate was 9,6% with all giving a result when repeated. The majority of mothers (92%) preferred POC testing with 7% having no preference. No staff preferred SOC testing with 79% preferring POC and 21% having no preference.

**Conclusions:**

Point-of-care HIV testing for EID is accurate, feasible and acceptable, with benefits of early ART for all positive infants at birth facilities. We recommend that it be considered best practice for EID.

**Trial registration:**

ISRCTN38911104 registered 9 January 2018 – retrospectively registered.

**Electronic supplementary material:**

The online version of this article (10.1186/s12889-019-6990-z) contains supplementary material, which is available to authorized users.

## Background

In the era of Sustainable Development Goals, Universal Health Coverage and Integrated People-centred Health Care, streamlined health systems and respectful care are necessary [1–3]. People–centred health care considers patients, healthcare workers and communities to make the best healthcare decisions within a health system. South Africa has more than double the HIV burden of any other country [4] and therefore innovative ways to improve service delivery and patient-care are required. Great strides have been made towards elimination of mother-to-child transmission of HIV (EMTCT) in South Africa with > 95% of pregnant women receiving antiretroviral therapy (ART) [5], resulting in a large reduction in mother-to-child transmission (MTCT). With an estimated transmission rate of 1,2%, translating to 384 HIV-infected infants per 100,000 live births [6], this is significantly higher than the 50/100000 target for EMTCT set by the World Health Organization (WHO). Even with EMTCT, the estimated 360,000 infants born to HIV-positive mothers in South Africa every year [7] will require HIV testing at least twice in their first year of life with the birth and 10-week testing schedule.

Early infant diagnosis (EID) of HIV offers the opportunity to commence early ART which is associated with low viral reservoirs [8] and provides the best prognosis for HIV-infected infants [9, 10] with possibilities of remission [11, 12]. Challenges in EID programmes in many countries include missed or delayed testing, long turn around times (TAT), failure to get results, duplicate testing, early mortality peak in HIV-infected infants, poor linkage to care and loss to follow-up (LTFU) [13–22].

Cepheid® and Alere™ both have point-of-care (POC) EID platforms that are prequalified by the WHO with the Real World Diagnostics platform - Simple Amplification-Based Assay (SAMBA) awaiting prequalification. Evaluation studies of these platforms have shown similar accuracy to the centralised Roche COBAS Ampliprep/COBAS Taqman (CAP/CTM) HIV1 Qualitative Test (Roche Molecular Diagnostics, Brachburg NJ) testing [23–28].

A field evaluation study of the Cepheid Xpert HIV-1 Qualitative test showed median same-day TAT, 100% ART initiation of HIV-infected infants with reduced ART initiation time [29]. Implementation of the Alere™q HIV-1/2 Detect POC EID test in Malawi and Mozambique showed markedly increased infant ART initiation [30, 31] with improved retention of HIV-infected babies in ART care after receiving POC testing in the Mozambican study [31].

The Elizabeth Glaser Pediatric Aids Foundation (EGPAF) and UNITAID are funding the implementation of enhanced EID testing into 9 African countries, which includes POC testing to strengthen poorly performing EID programmes. With conventional EID, 50% of results reached the caregiver and 51% of positive children are commenced on ART whereas with POC EID 99% of care givers receive the result and 93% of infants are commenced on ART [32].

South Africa has the largest EID program in the world with routine polymerase chain reaction (PCR) HIV testing of exposed infants at birth, 10 weeks and 6 weeks post breastfeeding cessation. EID coverage is reported at > 95% [5]. This amounts to approximately half a million tests performed annually at regional central laboratories [33]. Many of the same gaps are experienced with missed testing [22], decreased 10-week testing [34] sample collection error, clerical errors, pre-analytical and analytical lab errors [33]; and it is unknown how many mothers actually get the HIV results. Each dried blood spot (DBS) specimen taken for EID requires the following 10 steps to get the result to the health care worker (HCW) and then the patient.

1. Correct labeling of specimen
2. Correct labeling of request form
3. Sufficient uncontaminated sample
4. Lab barcode into patient held Road To Health Card (RTHC)
5. Transport to 2 laboratories
6. Correct logging into each laboratory
7. Accurate testing giving a conclusive result
8. Result hardcopy to clinics or functioning computers and operators at clinics
9. TAT < 3-6d so as to be accessed at 1st postnatal visit
10. HCW to remember to access result at clinic and write in RTHC

From anecdotal experience, errors had been encountered at each step.

It was therefore hypothesised that this process could be done by one HCW at one time point with real time quality control (QC) averting the 6,2% missed diagnostic opportunities reported by the laboratory services [33] and the possible 9,8% unnecessary repeat testing [35] and the frustrations for mothers and HCWs with delays in appropriate clinical management. A recent presentation at CROI showed that in South Africa, 50.8% of HIV-infected infants between 2010 and 2015 were only diagnosed after 3 months of age (median 8 months) due to late presentation suggesting poor access to care for these high-risk mothers and infants [35].

Given the need for more local South African data this implementation study aimed to evaluate the accuracy, feasibility and acceptability of implementing the Alereq HIV 1/2 Detect POC EID test into a hospital and primary health care (PHC) clinic in Durban, the city with the highest HIV burden in South Africa with a reported antenatal prevalence of 44% [36].

## Methods

### Objectives

The primary objective was to assess the accuracy of POC testing compared to the central laboratory standard of care (SOC) testing. The secondary objective was to assess the feasibility and acceptability of implementing POC HIV PCR testing in a hospital and PHC clinic.

### Study design, site and population

An effectiveness-implementation hybrid type-2 study was undertaken in Durban, South Africa. The study population consisted of HIV-exposed infants presenting for HIV-PCR testing at birth at Addington Hospital (a regional hospital in the city centre) and follow-up testing at a referral PHC clinic, Lancers Road Clinic, (in the transport hub of Warwick triangle taxi rank).

### Pre-implementation situational analysis

Prior to commencing the implementation of POC testing, a situational analysis was undertaken at Addington Hospital from July 2016 to January 2017 prior to the intervention to collect baseline data on EID of HIV-exposed infants from the post-natal ward. PMTCT program data including contact information was collected from the postnatal register and entered into Microsoft Excel and mothers were contacted telephonically to enquire about their EID testing and the spreadsheet updated.

### Intervention - Alere™q HIV-1/2 detect testing

The implementation of the Alere™q HIV-1/2 Detect point-of-care RNA PCR test was performed for HIV-exposed infants concurrently with the SOC central laboratory DBS test (COBAS® AmpliPrep/COBAS® TaqMan® (CAP/CTM) HIV-1 Qualitative test v2.0 (Roche® Molecular Systems, Inc., Branchburg, New Jersey, United States). Results were given from both tests.

The Alere platform was chosen due to its ease of use with no sample preparation steps and 50 minutes (min) to test result with no additional computer required. The Alere™q is a portable bench top fully automated PCR instrument with a power drum giving it an 8-h battery life. A drop of blood (25-50 μl) is collected directly into the capillary of the Detect cartridge, which is loaded into the instrument as soon as possible (within 10 min of collection). The sample is processed in 50 min by the Alereq instrument after which the result is printed using the provided thermo-printer and written and stapled into the infants Road to health card (RTHC). Results are also transmitted to Alere Datapoint via a supplied modem and can then be accessed remotely. Two POC instruments were made available for the study by Alere™ (now Abbot Point of Care).

### Implementation phase

The POC intervention was implemented first at Addington Hospital from February to June 2017 and then at Lancers Clinic from June to August 2017. Point of care PCR testing was performed during office hours (Monday – Friday) when study staff was available to obtain consent and administer questionnaires.

#### Study cohort

**Fig. 1:**
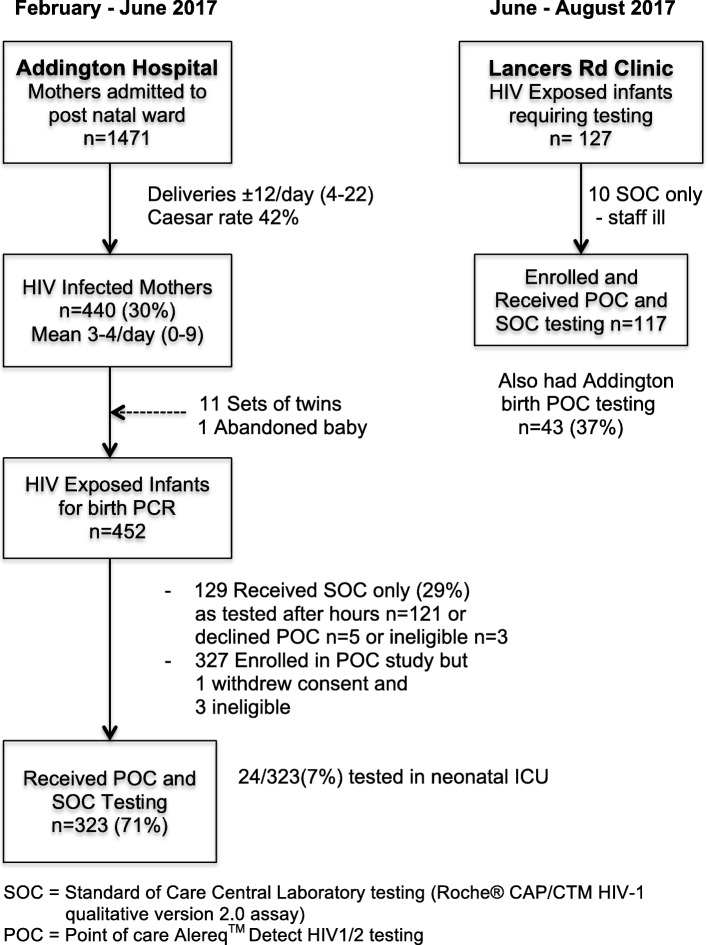
Cohort Profile at Hospital and Clinic. HIV-exposed infants were enrolled for POC PCR HIV testing for birth testing at Addington Hospital and follow-up testing at Lancers Rd. Clinic to compare with routine laboratory SOC testing

All infants of HIV-positive mothers were eligible if their mother consented to participate in the study. At Addington Hospital, 323 (71%) of the HIV-exposed infants received POC and SOC testing. Three mothers declined all PCR testing and on further counselling agreed to SOC testing. Another mother declined the study and 121 mothers received after hours SOC testing only. Of the 327 mothers who enrolled in the study, one withdrew consent after an error POC test when it was discovered that the SOC test result was available and three were found to be underage and therefore ineligible. Twenty four (7%) tests were performed on infants in the neonatal ICU. At Lancers Clinic 117 (92%) of the HIV-exposed infants received POC and SOC testing (Fig. 1).

#### Data collection methods

All mothers signed voluntary informed consent for the study and a study specific ‘birth questionnaire’ (Additional file 1) was administered to Addington mothers and a study specific ‘10 week questionnaire’ (Additional file 2) recording socio-demographic data, history, preferences and acceptability. Another study specific ‘staff questionnaire’ (Additional file 3) was administered to all clinical staff performing the POC testing at the end of the study recording their experiences, preferences and acceptability of POC testing. Questionnaire data was entered into EpiData. (EpiData Association, Denmark). Laboratory data was captured in Microsoft Excel. PMTCT program data continued to be collected from the ward register and entered into Microsoft Excel.

#### Implementation procedures

The POC instrument was placed in the well-baby nursery in the postnatal ward at the hospital where mothers and infants were brought for pretest counseling and PCR testing as per National Guidelines [37]. Similarly, the POC instrument was placed in the well-baby examination room at the PHC clinic and, as mothers and babies presented for their clinic visit, they were pre-test counseled, consented and the PCR testing performed. Post-test counseling was conducted with all mothers after results were available ensuring privacy and confidentiality. All POC test errors were repeated until a result was obtained. For testing infants admitted to the neonatal intensive care unit (ICU), the Alereq instrument was initially transported to the neonatal ICU. It was later found to be preferable to take the specimen from the neonate in ICU and transport the cartridge down 2 floors to the POC instrument in the well baby nursery as this took under 10 min (per manufacturers specifications). Hospital, clinic and research staff involved in infant testing all completed and passed the Alereq end-user training course. Research staff performed all the DBS specimen collection for testing on the study infants over the intervention period to allow hospital and clinic staff to focus on performing the POC test without increasing their workload. Post-test counseling was conducted with all mothers on the day of testing and if the infant was found to be positive, confirmatory testing was performed and infants commenced on ART by the hospital Paediatric team.

### Statistical methods

Sample size calculations were performed to address the primary objective of assessing the specificity of the EID test compared to SOC, considered the gold standard. To detect a specificity rate of 98% within a 1.3% margin of error at Type 1 error rate of 5%, 438 HIV-negative babies were required. Assuming that the prevalence of HIV in babies at birth or 10 weeks lies between 1 and 3%, we were required to screen 452 babies for inclusion in the study. Statistical analysis was performed using Stata 14. Continuous variables were summarized as means (with standard deviations) or medians (with interquartile ranges), where appropriate. Categorical variables were described using frequencies and percentages. A content analysis was performed for staff and mothers’ responses to questions on acceptability and preference with manual coding of manifest content and quantitative analysis.

## Results

### Pre-implementation situational analysis

During baseline data collection 258 mothers who delivered HIV-exposed infants in the first 10 weeks of the situational analysis were telephoned to follow up on their babies’ HIV testing. They were called 4–6 months after the birth of their child. Only 102 of the 258 (40%) could be contacted with the phone numbers recorded at the hospital (3 call attempts on 3 different days). Seventeen percent (17/102) reported not receiving their baby’s birth result, 19% (19/102) reported not having a 10-week test and 31% (32/102) had not received the 10-week test result. Over the entire 7 months prior to the intervention there were 765 HIV-exposed live infants born at the hospital. Thirteen of the 765 (2%) were not tested in error, 6 (1%) died in hospital before being tested, 6 (1%) were HIV-infected, 730 (95%) were HIV-negative, 10 (1%) had no result due to lab errors and 43 (6%) were logged incorrectly on the computer system so that the result was not available with the barcode in the Road-to-Health Card. This meant that 66 (9%) could be found to have no result at the clinic if identified, with 23 (3%) requiring testing and 43 (6%) being retested in error due to clerical laboratory issues. This problem was highlighted to the hospital and central laboratory on repeated occasions but continued to occur on and off throughout the study.

### Implementation phase

**Fig. 2:**
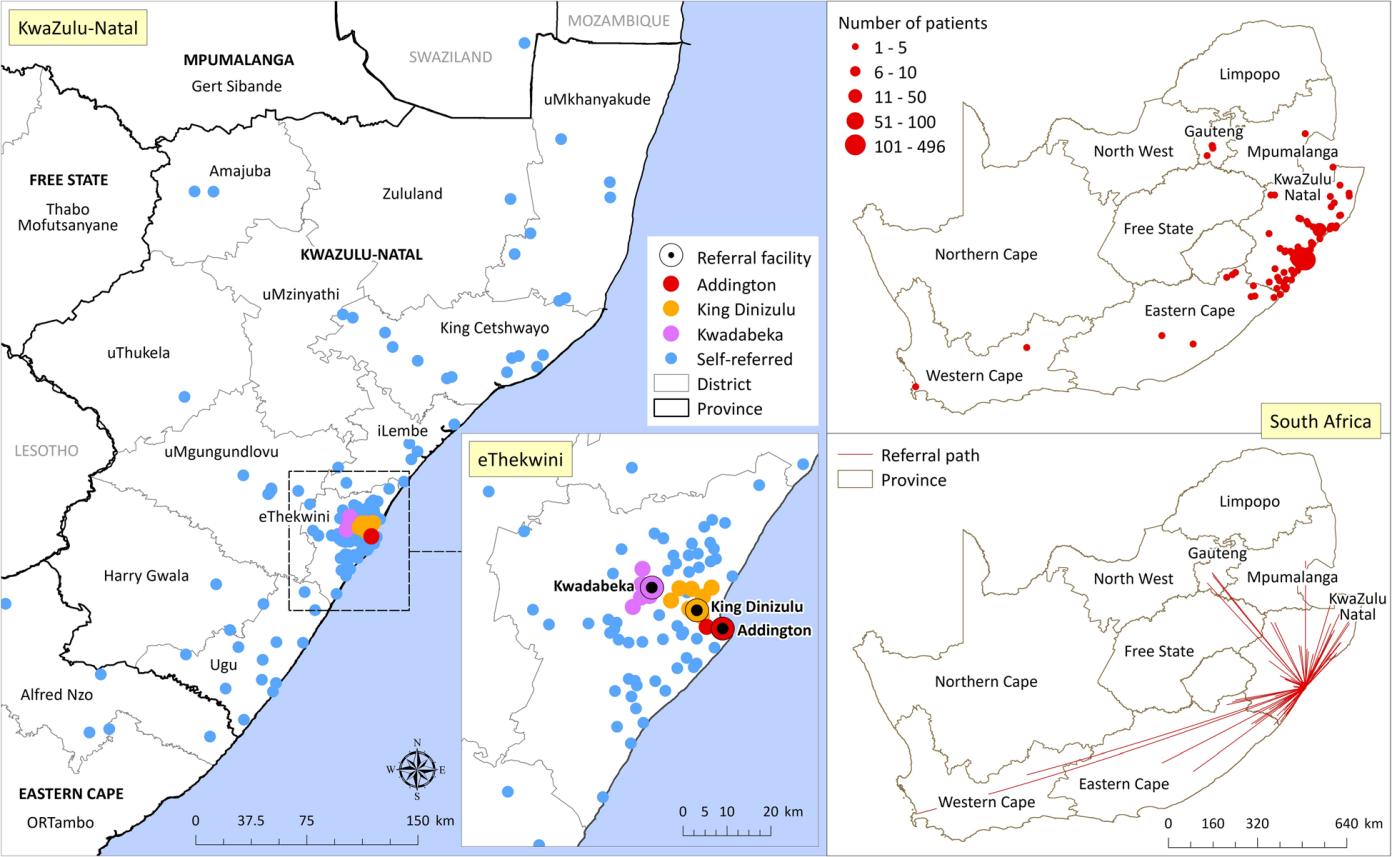
Maps of Antenatal booking clinics. Points on maps represent the first clinics where pregnant mothers booked before coming to deliver at Addington hospital during the study period (Feb-June 2017) as recorded in their antenatal record book. Four patients with incomplete facility information have been referenced to center point locations of the province, town or village. All maps represent the same data in different ways to highlight the mobility of mothers in this context

Over the duration of the study intervention at Addington Hospital (February to June 2017), a total of 1471 mothers delivered live infants with 440 (30%) of mothers being HIV-infected. The antenatal booking clinics shown in Fig. 2 demonstrate the mobility of mothers from pregnancy booking to delivery. With Addington Hospital being a Regional Hospital it delivered infants of mothers booked at Addington Hospital antenatal clinic (ANC) *n* = 341 (23%) and Lancers Clinic *n* = 496 (34%) and received referrals from other birthing facilities and their clinics in Durban, including King Dinizulu Hospital and its clinics *n* = 152 (10%) and Kwadabeka Community Health Centre and it’s clinics *n* = 195 (13%). The remaining 13% of mothers (*n* = 192) come from other non-referral clinics, 3% were unbooked (*n* = 47) and 3% (*n* = 48) had their booking clinics not recorded in the register.

#### Socio-demographic data

Socio-demographic characteristics of participants are represented in Table 1.

**Table 1:** Socio-demographic Characteristics of HIV-infected Mothers and their babies at both study sites

	Addington Hospital max *n* = 323	Lancers Rd. Clinic max *n* = 117
1. BABIES (A:323 L:117)^a^		
Male n (%)	167 (52%)	57 (49%)
Birth weight (A:322 L:117)^a^		
BW ≥2.5 kg n (%)	277 (86%)	105 (90%)
LBW < 2.5 kg n (%)	40 (12%)	11 (9%)
VLBW< 1.5 kg n (%)	5 (2%)	1 (1%)
2. MOTHERS (A:322 L:115)^a^		
Age of mothers, mean (range)	29 (18–43)	30 (19–42)
Maternal ART history (A:316 L:115)^a^		
On ART before pregnancy n (%)	162 (51%)	58 (50%)
Median duration on ART, months (IQR)	33(19–56)	45(19–60)
Started ART this pregnancy n (%)	154 (49%)	57 (50%)
Median duration ART, months (IQR)	4(3–6)	8(6–10)
ART Regimen (A:298 L:117)^a^		
On 2nd line therapy n (%)	12 (4%)	2 (2%)
Not on ART prior to Delivery n (%)	7 (2%)	2 (2%)
Highest Education (A:311 L:116)^a^		
None	12 (4%)	0
Completed Primary n (%)	24 (8%)	6 (5%)
Completed Secondary n (%)	129 (41%)	38 (33%)
Tertiary n (%)	70 (23%)	33 (29%)
Current Partner (A:322 L:117)^a^		
No current partner n (%)	13 (4%)	11 (9%)
Partner HIV status unknown n (%)	125 (40%)	32 (30%)
Partner HIV known negative n (%)	54 (29%)	23 (32%)
Partner HIV known infected n (%)	130 (71%)	50 (68%)
Partner, if infected on ART n (%)	100 (78%)	35 (70%)
Dwelling (A:321 L:117)		
Formal n (%)	277 (86%)	108 (92%)
Informal n (%)	44 (14%)	9 (8%)
Employment:(A:322 L:117)^a^		
Full time n (%)	95 (30%)	52 (44%)
Part time n (%)	49 (15%)	21 (18%)
Self-employed n (%)	19 (6%)	6 (5%)
Unemployed looking for work n (%)	48 (15%)	13 (11%)
Unemployed not looking n (%)	73 (23%)	19 (16%)
Student/scholar n (%)	38 (12%)	6 (5%)
Income (A:311 L:115)^a^		
Receiving any welfare grants n (%)	176 (57%)	73 (64%)
Monthly household income, median ZAR (range)	3000–5000	3000–5000
Declined to answer	20 (6%)	1 (1%)
Didn’t know household income	160 (51%)	42 (37%)

^a^denominators depending on data available. A = Addington Hospital, L = Lancers Clinic

The cohorts were similar across both sites with half of mothers commencing ART before this pregnancy. Their partner’s HIV status was unknown by 30–40% of mothers and nearly one third reported that their partners were negative.

Across both sites 5% (21/422) of mothers were reported to be foreign nationals from other African countries. For 21% (89/429) of mothers this was their first child (primups), 55%(179/326) of mothers had biological children not living with them and 2%(6/318) had other HIV-infected children. Most mothers chose to exclusively breastfeed at birth - 79%(88/111) in the Lancers cohort and 91%(285/314) in the Addington cohort with Addington Hospital being a WHO/UNICEF Baby-friendly Hospital. Eighty-three percent (257/309) of the Addington and 90%(90/100) of the Lancers mothers had never been legally or traditionally married. Antenatal data from the Addington cohort included 5%(16/321) being unbooked and 57%(175/305) booking before 20 weeks gestation (calculated back from their expected date of delivery) with 81%(260/322) of mothers having one or more ultrasound examinations in pregnancy. The Caesarean section rate was 49.5%(160/323) for the study cohort in keeping with it being a regional referral hospital. The median CD4 count for mothers was 448 cells/μl (IQR 308–582 cells/μl).

**Fig. 3:**
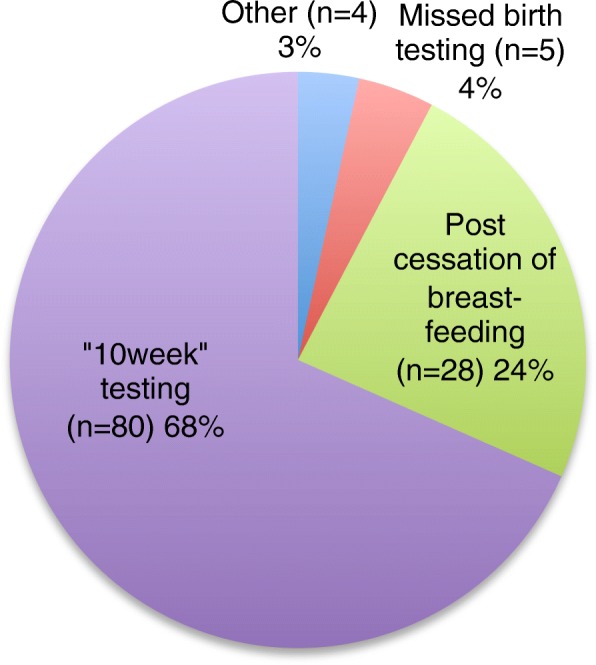
Lancers Clinic indications for HIV-PCR testing *n* = 117. HIV-exposed infants presented to a PHC clinic for HIV testing according to various indications. The majority were for follow-up confirmatory testing after a birth test usually performed at the 10-week immunization visit with another substantial number requiring testing 6 weeks after stopping breastfeeding as per PMTCT guidelines

All EID testing at Addington was birth testing and the indications for EID testing at Lancers Clinic were missed birth testing 4% (*n* = 5) from private hospitals, ‘10-week’ follow-up testing 68% (*n* = 80), post breastfeeding cessation 24% (*n* = 28) and 4 others (3%) for seroconversion of mother post-delivery, repeat of indeterminate birth test, mothers request and clinically indicated (Fig. 3).

#### Test results

Addington POC testing yielded 318 (98.5%) negative and 5 (1.5%) positive results. Four mothers (1%) were known to have seroconverted during pregnancy and all their infants had negative birth PCR results. All HIV-infected infants had confirmatory testing (3 POC confirmations and 2 had previous positive SOC tests) and all were commenced on ART before discharge from the hospital. Intervention group SOC testing yielded 316 negative results with 1 specimen discarded in error and 1 test missed in error. Subsequent SOC testing for the two missing SOC tests, at 6 weeks and 1 week respectively, yielded negative results. Six specimens were logged at the lab incorrectly so could not be found with barcode. Of the five positive POC results, four correlated initially with a positive SOC result, while one had an indeterminate result. The infant was retested after 4 weeks on ART and remained indeterminate on SOC PCR testing with a viral load detectable at less than 150 copies per ml, which was below the assay’s limit of quantification. Subsequent virological testing was performed on this infant at the central research laboratory and after 5 months of ART exposure peripheral blood mononeuclear cells (PBMCs) were isolated with 1/40 positive for HIV DNA on nested PCR. This set of results suggests likely HIV infection albeit with a low viral reservoir. The patient continues to be managed on ART with close virological monitoring. On telephonic follow-up of the 5 positive infants at 5–6 months one could not be contacted, 3 reported that their infants were on ART at other facilities and one was in care at Addington hospital.

Lancers Clinic POC testing yielded 117 negative results. SOC yielded 116 negative and one invalid result which when queried by the researchers should have been re-tested and was found to be negative.

There was therefore ultimately excellent correlation between POC and SOC testing (*n* = 440) with the 435 negatives and 5 positives across both sites with specificity and sensitivity of 100%, but 10 SOC results required the researchers intervention to ensure a result.

Of the 29% who received SOC only at Addington hospital during the study period (*n* = 129), 1 infant was discovered to be positive when the infant presented ill at 6 weeks in the paediatric ward. Six infants were not tested in error, 1 reported a lab error, 1 was indeterminate, and 2 specimens logged incorrectly resulting in 8/129 (6%) not receiving a birth result and 2 may have been repeated unnecessarily as results were difficult to find.

Turn-around-times (TAT) for results were 0 days for POC tests with all mothers receiving the results on the same day as testing with a median of 8 days for the SOC testing (Interquartile range (IQR) 6–10 days; range 3–22 days). Mothers are required to attend a postnatal check-up visit at their local clinic at 3–6 days post-delivery. Therfore only 25% may have been able to receive their birth PCR result at this visit.

The Alereq Detect error rate was 9,6% (47/490) tests performed with 2 tests repeated twice to yield a result. Error rate was 10,0% (38/364) at Addington Hospital with 21 different operators performing testing and 7,6% (9/126) at Lancers Clinic with 7 operators performing testing. Error types were sample detection 2 (4%); cartridge 1 (2%); device 31 (66%); control 6 (13%) and analysis 7 (15%). When a POC instrument gave 3 consecutive errors, Alere field support was contacted and responded within a day with one instrument being replaced within a week (during which only one instrument was operational) and the other having a software upgrade on site that corrected the problem. The POC instruments performed well though the Durban summer heat with ambient temperatures up to 37 °C with no air-conditioning at either study sites. Although 2 POC instruments were used, one instrument would be sufficient if implemented as it would be able to operate 24 h a day in the hospital setting (not just 8 h per day as during the study).

Our observational assessment is that no additional staff would be needed to add POC testing into the hospital and clinic setting for daily operating after health care workers are adequately trained. However, administrative oversight would be needed for stock control, quality control, data management and technical support which could be offered by the Alere field support service and the central laboratory services.

#### Maternal viral load monitoring

**Fig. 4:**
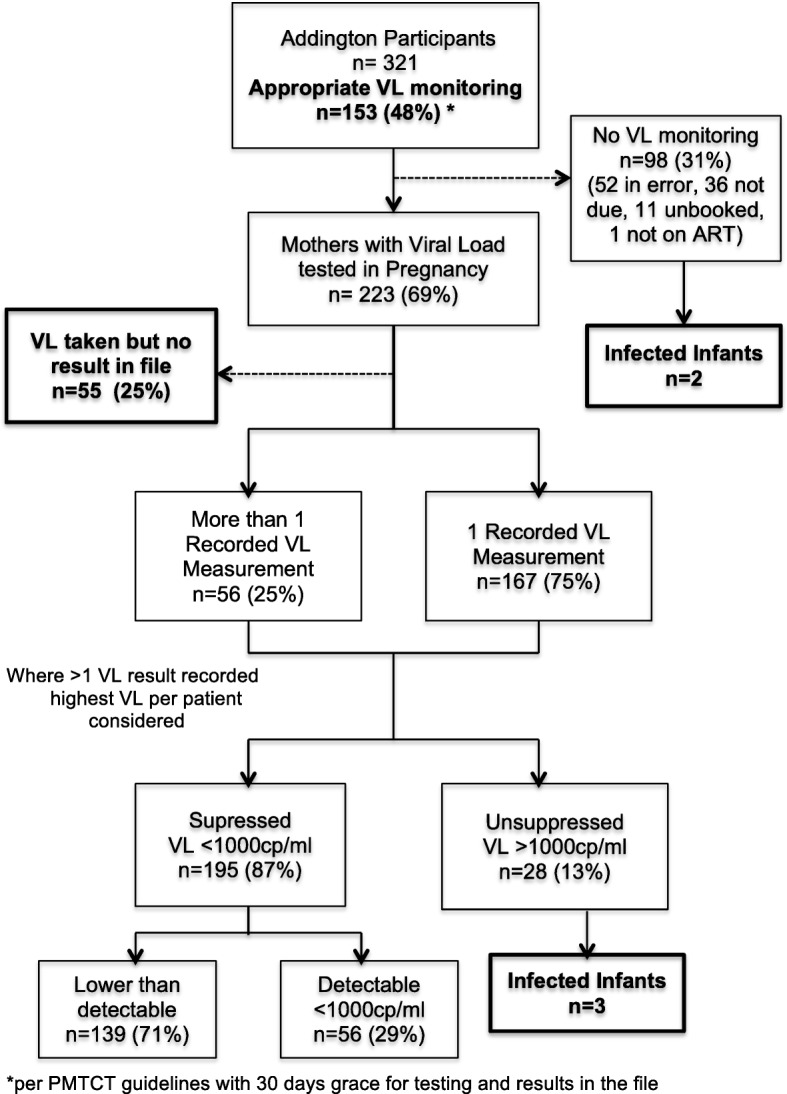
Maternal Viral load results during pregnancy from ANC files. Viral load monitoring of the HIV-infected mothers of infants that had POC HIV testing for the study is detailed. Results were extracted from their ANC charts and NHLS LabTrack database. No infected infants had mothers with recorded suppressed viral loads and only 69% of mothers had a viral load test done but a quarter of those results were only found on the national database so not used clinically. Almost half were not monitored appropriately

Maternal viral load (VL) monitoring was assessed from the ANC files of 321 mothers at Addington (Fig. 4). Of the 16 unbooked mothers, 8 were on ART prior to delivery, 4 starting before pregnancy and 4 during pregnancy. Only 5 of the unbooked mothers had VL testing at admission for delivery, 4 of which were > 1000 copies/ml and one was 325 copies/ml. Viral load monitoring was reviewed according to current PMTCT guidelines, with a grace period of 30 days for testing and results to be in the file. Only 48% of mothers had appropriate VL monitoring with the results recorded in the ANC file. No results were recorded in the files for 25% of the VL tests found on the NHLS database. Of the 31% of mothers with no VL monitoring (*n* = 98), 51 (52%) were not done in error, 35 (34%) were not yet due, 11 were unbooked and not done at admission and 1 was not on ART. Of the 5 HIV-infected infants, 2 mothers had no VL monitoring, of which one reported defaulting her ART and one was not on treatment. The remaining three mothers of positive infants had known VL > 1000 copies/ml; (1 unbooked, 1 result was not in the file and one had TB and commenced ART 6 weeks before delivery).

**Fig. 5:**
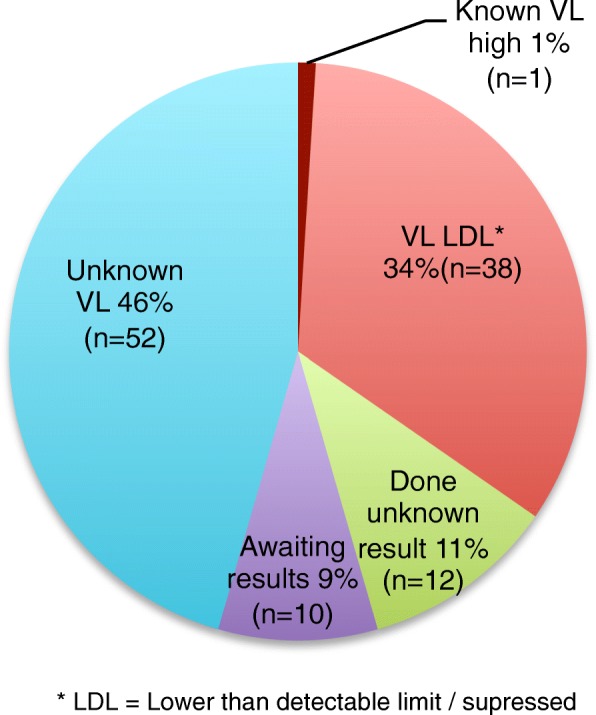
Lancers Clinic mother’s viral load report. HIV-infected mothers of infants tested with POC PCR for HIV at the clinic were asked if they knew their latest viral load result. Almost 50% were unaware of VL testing and 11% knew it was taken but did not know result

At Lancers Clinic, 115 mothers had reported VL information: 2 were not yet due for VL testing, 52/113 (46%) had no knowledge of VL monitoring with a subsequent 10/113 (9%) awaiting results and 12/113 (11%) not knowing the result. Suppressed VL results were reported by 38/113 (34%) and 1 knew her VL was high (Fig. 5).

#### Acceptability

The vast majority of mothers preferred getting their baby’s HIV PCR result on the same day (398/434–91,7%) and only 1,6% (7/434) preferred getting the result at a later date with 6,7% (29/434) having no preference. Mothers comments were summarised: “Happy” for 84% (365/434), “Good to know same day” for 12% (50/434), “Relieved/ free from stress” for 10% (42/434) with 14/434 (3%) wanting to be sure with SOC result and 2 not believing the results. Seven mothers (1,6%) commented that the government should use these machines. Of the mothers with HIV-infected infants, 3 preferred the same day result and 2 had no preference. One mother said she accepted the positive result, two were confused and 2 were very sad. Patients made more than one comment.

Hospital and clinic staff were encouraged to perform the POC testing and undertook 262/440 (60%) of infants’ POC testing. At Addington Hospital 174/323 (54%) infants’ POC tests were performed by Registered Nurses – 67 tests (39%), Enrolled Nurses – 82 tests (47%) and Enrolled Nursing Assistants – 25 tests (14%). At Lancers Clinic 88/117 (75%) of infants’ POC testing was performed by Enrolled Nurses - 35 tests (40%), an Enrolled Nursing Assistant − 8 tests (9%) and counsellors - 45 tests (51%). Study staff performed the remainder of the POC testing and all the SOC testing. All staff felt they were adequately trained with Alereq end user training and 4/24 said they had challenges initially but became accustomed to the POC instrument.

**Fig. 6:**
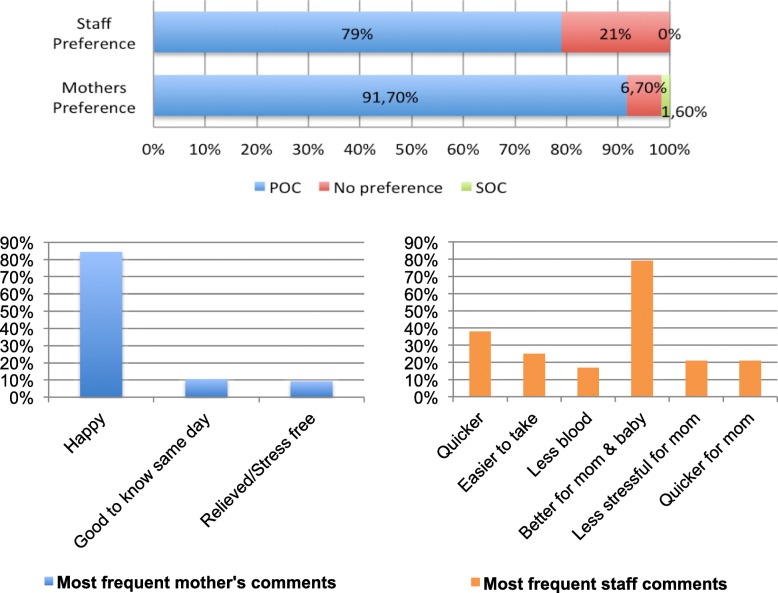
Staff and Mothers’ Preferences and Comments Summary. Mothers of HIV-exposed infants and staff performing testing were asked whether they preferred POC testing or SOC laboratory testing and asked to comment. Point of care testing was preferred by the vast majority of staff and mothers. Staff found the POC test quicker and easier to perform

The majority of staff 19/24 (79%) preferred POC testing with the remaining 5 having no preference between POC and SOC testing. Thus no staff preferred SOC testing. Staff comments were coded with 38% reporting POC sample collection “quicker” than DBS collection; 25% - “easier to take”; 22% - “less blood”; and 17% - “results quicker”. The majority of staff (92%) thought POC testing was better for the mother with collated comments being 79% - “better for mother to get result on same day”; 21% - “less stressful for mother”; and 21% - “quicker for mother”. The positive infants starting treatment at birth was mentioned as a strength of POC testing by 13% of staff. One staff member had concerns that it was too much for mothers to get result on the same day and one felt DBS testing per SOC was more reliable (Fig. 6).

No adverse events or harms were experienced at either study site by any mothers, babies or staff.

## Discussion

This study outlines many of the inter-relating factors involved in the care of HIV-infected mothers and their infants in this high prevalence setting and demonstrates the feasibility and acceptability of POC EID testing in a hospital and clinic setting. There was ultimately perfect correlation between POC and SOC testing, however the researchers had to actively follow up in 10 cases to secure the SOC results, which is unlikely to occur in common practice and would all be avoided with POC testing. These missed diagnostic opportunity tests were reported as only being repeated in one third of cases in routine care from SOC National Health Laboratory Services data by Mazanderani et al. [33].

From the situational analysis telephonic survey more than half of mothers (60%) could not be contacted which was concerning, with continuing drop offs in the collection of results and testing at subsequent time points. The maps (Fig. 2) highlight the mobility of mothers over their pregnancy and the expected potential problems with tracing and follow up. Mobility is common in the pregnancy and post-partum period and is a major determinant in linkage to care [38, 39].

The value of a conclusive result on the day of testing cannot be over-emphasized as delays in confirmation of infant HIV status can lead to LTFU and infant deaths while awaiting confirmatory testing [40]; when even single dose nevirapine prophylaxis can reduce the viral load to below the limit of detection in 38% of infants at 5 days [41]. The benefits of early ART to minimise the viral reservoir continue to be demonstrated [8]. With POC testing there may be value in revisiting the previous guidelines of baseline viral load testing in HIV-infected infants, performed on a sensitive platform to further confirm 2 positive POC tests and quantify the VL, as this VL correlates with the viral reservoir and future prognosis. This could have assisted with confirmation of the diagnosis in the infant who tested positive on POC and indeterminate by SOC. With subsequent viral suppression and potentially no seroconversion in these HIV-infected infants this initial diagnostic time-point is critical [42].

The post-test counselling to the vast majority of mothers with infants with negative tests was observed by the study staff to be a very valuable counselling touch-point as it was a happy positive occasion to celebrate with her, with a window to answer her questions; encourage disclosure and partner testing; reaffirm her ART adherence; discuss the meaning of VL monitoring and its importance; discuss infant feeding, infant prophylaxis administration and the need for further infant testing at 10 weeks and post breastfeeding. With evidence of viraemia and poor adherence [43, 44] during this time period, a counselling checklist could be helpful at this time-point.

Mothers of positive infants in the study had challenging and complex social situations, with 2 unbooked, 2 defaulting treatment and one with TB co-infection and a preterm infant, as has been documented in other studies [45–47]. Mothers with high VLs, are often not engaged or have disengaged with the healthcare system creating a vulnerable population in need of differentiated care. Initiating neonatal ART in a birthing unit/hospital environment was preferable as phlebotomy for baseline bloods and neonatal dosing of ART requires time and precision and difficulties were previously experienced by the investigators at a PHC clinic level. With the mother as an inpatient the additional time for counselling, support and family engagement is more readily available. There is more time to assist and monitor the mother with administering 3 syrups to her new baby twice a day.

The finding that 40% of the Addington Hospital mothers and 30% of the Lancers Clinic mothers did not know their partner’s HIV status reaffirms this high-risk population. However, of the partners known to be HIV-infected, 78% (Addington Hospital) and 70% (Lancers Clinic) were reported to be on ART. This reaffirms the benefits of disclosure as nationally only 47% of HIV-infected men were estimated to be on ART in 2016 [48].

High acceptability of POC testing by mothers and staff with the overwhelming majority preferring POC testing; 100% result to patient rate on the same day as testing; 100% initiation of HIV-infected infants on ART and the simplicity of the testing are highly in favour of POC testing. Staff’s time for POC and SOC testing were similar in total; while the POC sampling was much quicker (1–3 min for POC versus 10–12 min for SOC) the post-test counselling required for POC made them similar.

Despite South Africa’s wide ART coverage for pregnant mothers (97% in the Addington cohort), there is evidence of adherence barriers, viraemic episodes and reduced retention in care [43, 49–52]. At Addington, 25% of viral load test results were not in the patient files at delivery, which may never be accessed and so would then be completely wasted tests at great cost to the health-care system. It was concerning that 46% of the Lancers mothers on ART had no knowledge of VL testing, and 48% of Addington mothers did not have appropriate VL monitoring as per PMTCT guidelines. This highlights the need for active engagement of staff and patients in VL monitoring. If a test result is never accessed it is a waste of resources. Watching blood going into a ‘machine’ with rapid results would facilitate awareness of the importance of VL results as well as real-time management. With antenatal and postnatal services being positioned together as maternal and child health (MCH) in the ‘Ideal clinic’ PHC model being rolled out in South Africa, one POC VL/EID instrument could augment PMTCT at a clinic level with ‘real-time’ management of mothers and babies and appropriate differentiated care. Maternal VL testing at birth is now a guideline in South Africa so if it could be performed on the same POC platform as the EID test in the postnatal ward this would again strengthen the PMTCT service, engage the mother and staff in her care and create awareness of the value of VL monitoring. Real time VL monitoring would allow differentiated care [53] with targeted interventions for this vulnerable population of mothers with high VLs and closer monitoring of their infants.

POC testing is not a solve-all intervention but it provides patients results for response in real-time and eliminates some of the current challenges [54] and there is evidence of additional POC tests being requested by clinic staff [55]. This allows time and resources to be channelled into the other many challenging areas of patient treatment and adherence; monitoring; counselling and support; and LTFU [43, 56, 57]. POC testing can thus enhance the effectiveness of current programmes [58]. With POC results able to be transmitted centrally via the Alere Data-point platform they could be linked to the laboratory information management systems for quality management and access at other facilities.

Despite South Africa’s wide EID coverage of > 95% [5], POC EID testing would further strengthen and streamline the massive screening process (over 500,000 tests required per annum) and assist with rapid ART initiation and linkage to care for HIV-infected infants. UNICEF has released a toolkit for introducing POC diagnostics technologies into national health systems due to the laboratory-based technologies not meeting the needs of the HIV epidemic [59] but is it not time to think beyond the laboratory paradigm [60], as even with a good laboratory service in South Africa, many gaps remain.

### Study limitations

This implementation was only conducted at one hospital and clinic, which may not be representative of the whole province or country. Lack of concurrent POC maternal VL testing is also a limitation of this study. Lack of a control group or randomisation is a further limitation of the implementation assessment. Much of the socio-demographic data is reported by participants when questioned so could be biased. The low number of HIV-infected infants does not allow a significant assessment of the sensitivity of the POC PCR test. Experienced research staff performed all SOC specimen collection, which could have contributed to the fewer missed diagnostic opportunities than previously reported [33]. While cost effectiveness comparisons would be useful, it is difficult to put a monetary value to the many qualitative aspects of this process. HIV-positive mothers and their infants are a very defined population within which the need for infant HIV testing is universal suggesting generalisability. The challenges and logistics of EID experiences from many low and middle income [13, 18, 21, 61, 62] countries suggest that with adequate technical support; monitoring; and evaluation; as per UNICEF recommendations [59], our recommendation of best practice would also be generalisable.

To our knowledge, this is the first implementation of POC EID testing as true POC testing with heel-prick sampling, utilising clinical staff and initiating ART on POC results in South Africa.

## Conclusions

Testing HIV-exposed infants with a POC PCR test allowed for same day treatment initiation of the 5 infants who tested positive and same day results and post-test counselling for all the mothers of HIV-negative infants.

With its high accuracy, feasibility and acceptability to mothers and staff in this hospital and clinic setting and with the experience and benefit of POC EID demonstrated in the 9 countries in Africa [32], we recommend that POC PCR testing be considered as best practice for EID of HIV in exposed infants.

“Putting people at the centre of health care means ensuring that their voice and choice influence the way in which health services are designed and operate.” World Health Report (2008)

## Additional files

Additional file 1: Birth Questionnaire. (DOCX 463 kb)

Additional file 2: '10 week' Questionnaire. (DOCX 471 kb)

Additional file 3: Staff Questionnaire. (DOCX 432 kb)

## Abbreviations

ANC: Ante-natal clinic
ART: Antiretroviral therapy
DBS: Dried blood spot
EGPAF: Elizabeth Glaser Pediatric Aids Foundation
EID: Early infant diagnosis
EMTCT: Elimination of mother to child transmission
HCW: Health care worker
HIV: Human immunodeficiency virus
ICU: Intensive care unit
LTFU: Loss to follow up
min: Minutes
MTCT: Mother to child transmission
PCR: Polymerase chain reaction
PHC: Primary health care
PMTCT: Prevention of mother to child transmission
POC: Point-of-care
QC: Quality control
RTCH: Road to Health Card
SOC: Standard of care = Roche COBAS Ampliprep/COBAS Taqman (CAP/CTM) HIV1 Qualitative Test (Roche Molecular Diagnostics, Brachburg NJ)
TAT: Turn around time
WHO: World Health Organization
