# Supplementary material for: Point-of-care HIV testing best practice for early infant diagnosis: an implementation study
Source: BMC Public Health. 2019 Jun 11;19:731. doi: 10.1186/s12889-019-6990-z (PMC6560857; doi:10.1186/s12889-019-6990-z)
Supplement: Supplementary file 1 — Birth Questionnaire. (DOCX 463 kb) [file 12889_2019_6990_MOESM1_ESM.docx]

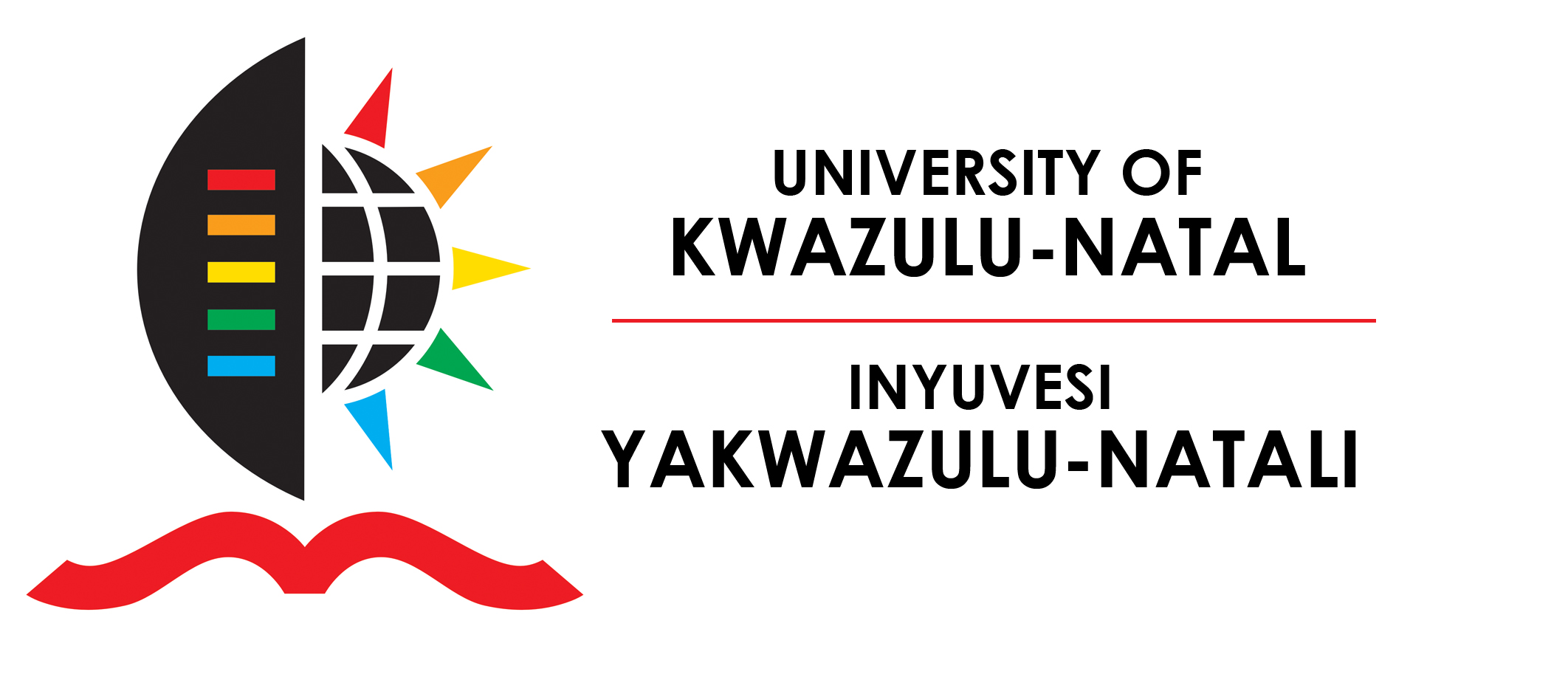

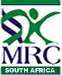
**Birth Questionnaire**

**Study no. _______________ Date: ________________**

**We would like ask you some background questions that are important for the study. All your information is confidential and the people reading the results will never know whom you are. You do not have to answer if you are not comfortable.**

**1. Age of mother** 1.1☐☐ years 1.2 DOB yy/mm/dd ☐☐/☐☐/☐☐

1.3 Are you a South African Citizen? Yes ☐ No ☐ Nationality _____________________

**2. Baby (pg 22, pg 27, pg 31) Clinic :_________________________**

2.1 Gender: M☐ F☐

2.2 DOB dd/mmm/yy ☐☐/☐☐/☐☐ 2.3 Time of delivery ☐☐:☐☐

2.4 Mode of delivery: NVD ☐ Breech ☐ Twins ☐ Caesar ☐ vacuum/forceps ☐

Comments/complications __________________________________

________________________________________________________________________________________________

2.5 Birth weight ☐☐,☐☐kg 2.6 Head Circumference (HC) ☐☐,☐cm

2.6 Apgar score 1min ☐☐/10 5min ☐☐/10

2.7 Feeding: Exclusive Breast ☐ Mixed ☐ Formula ☐

**3. ANC record**

3.1 Date of ANC booking? (pg 2) dd/mmm/yy ☐☐/☐☐/☐☐

3.2 LNMP? (pg 2) dd/mmm/yy ☐☐/☐☐/☐☐

3.3 Expected date of delivery EDD from dates? (pg 2) dd/mmm/yy ☐☐/☐☐/☐☐

3.4 Ultrasound date? (pg 2) dd/mmm/yy ☐☐/☐☐/☐☐

3.5 Expected date of delivery EDD from US? (pg 2) dd/mmm/yy ☐☐/☐☐/☐☐

3.6 Ultrasound Comments: ___________________________________

________________________________________________________________________________________________

3.8 Date HIV first diagnosed? (ask patient) dd/mmm/yy ☐☐/☐☐/☐☐

3.9 Date commenced ART? (ask patient) dd/mmm/yy ☐☐/☐☐/☐☐

3.10 Current ART regimen? FDC ☐ Other__________________________________________

3.11 Current CD4 count __________ 3.12 Hb_________ 3.13 Creat __________

3.14 RPR/Syphilis Positive ☐ Negative ☐

if Positive - Treatment given? Yes ☐ No ☐ - 3 Doses? Yes ☐ No ☐

**3.15 Viral load** results in pregnancy? Yes ☐ No ☐

(Record any results with dates and barcodes/MRN numbers available)

Date:_________ VL_________________ Barcode/MRC/CA # _____________

Date:_________ VL_________________ Barcode/MRC/CA # _____________

Date:_________ VL_________________ Barcode/MRC/CA # _____________

3.16 Any additional comments about pregnancy from file or patient? ________________________________________________________________________________________________________________________________________________________________________________________________

**4. Nevirapine/AZT**

4.1 Has your baby been given NVP yet? Yes ☐ No ☐

If yes what time was the first dose given? ☐☐:☐☐ am / pm

**5. Education**

| None |  | Some Secondary/High school |  |
| --- | --- | --- | --- |
| Some Primary |  | Secondary/High School completed |  |
| Primary completed |  | Tertiary/ After school |  |

**6. Partner**

6.1 Do you have a partner at the moment? Yes☐ No☐ if no go to **7**

6.1.1 Do you know his/her HIV status? POS ☐ NEG ☐ UNK☐

6.1.2 If positive… on ART? Yes ☐ No ☐ UNK ☐

6.1.3 Are you married? Yes ☐ No ☐ Divorced ☐ Widowed ☐

6.1.4 if Yes legal ☐ or traditional ☐

**7. Household**

7.1 Could you indicate the type of dwelling that you occupy at the moment?

| Dwelling/House or brick structure on a separate stand or yard or on farm | A |
| --- | --- |
| Traditional dwelling/ Hut/ Structure made of traditional material | B |
| Flat or apartment in a block of flats | C |
| Town house /semi-detached house in a complex | D |
| Semi detached house | E |
| House/Flat/room in backyard or in a house | F |
| Informal dwelling/Shack in backyard | G |
| Informal dwelling/Shack not in backyard, e.g. in an informal/squatter settlement or on farm | H |
| Caravan/Tent | I |
| Other, specify ………………………………………… | J |

7.2 How many live in your household? (including you)

Adults :- ☐ ☐ Children : ☐☐

7.3 Do you have other children that you gave birth to? Yes☐ No☐

6.5.1 If so, please complete the table?

| ChildNo. | Year born? | Stay with you?  Yes or No | HIV Status?  Pos, Neg, UNK | On ART?  Y, N, UNK |
| --- | --- | --- | --- | --- |
| 1 |  |  |  |  |
| 2 |  |  |  |  |
| 3 |  |  |  |  |
| 4 |  |  |  |  |
| 5 |  |  |  |  |
| 6 |  |  |  |  |

Food Security

7.4 In the past [4 weeks/30 days], was there ever no food to eat of any kind in your house because of lack of resources to get food?

Yes ☐ No ☐ (if no skip to 6.7)

7.4a How often did this happen in the past [4 weeks/30 days]? (Circle)

1 = Rarely (1–2 times) 2 = Sometimes (3–10 times) 3 = Often (more than 10 times)

7.5 In the past [4 weeks/30 days], did you or any household member go to sleep at night hungry because there was not enough food?

Yes ☐ No ☐ (if no Skip to 6.8)

7.5a How often did this happen in the past [4 weeks/30 days]? (Circle)

1 = Rarely (1–2 times) 2 = Sometimes (3–10 times) 3 = Often (more than10 times)

7.6 In the past [4 weeks/30 days], did you or any household member go a whole day and night without eating anything at all because there was not enough food?

Yes ☐ No ☐ (if no Skip to 7)

7.6a How often did this happen in the past [4 weeks/30 days]? (Circle)

1 = Rarely (1–2 times) 2 = Sometimes (3–10 times) 3 = Often (more than 10 times)

**8. Employment**

Which of the following best describes your present work situation?

| a.Employed full time | A |
| --- | --- |
| b.Employed part time | B |
| c.Employed less than part time (casual work/piecework) | C |
| d.Self Employed | D |
| e.Temporarily sick / Maternity leave | E |
| f.Unemployed, not looking for work | F |
| g.Unemployed, looking for work | G |
| h.Student/learner | H |
| i.Permanently sick or disabled | I |
| j.Volunteering | J |
| k.Other (specify) ………………………………………………………………….. | K |

**9. Income**

**9.1 Do you or anyone in your household receive any of the following welfare grants?**

| GRANT |  | Amount? |
| --- | --- | --- |
| a. No-one in household receiving any benefits | A |  |
| b. Old Age Grant / Pension | B |  |
| c. Child Support Grant | C |  |
| d. Disability Grant | D |  |
| e. Care dependency grant | E |  |
| f. Foster care grant | F |  |
| g. Grant in aid | G |  |
| h. Don’t know the name of grant | H |  |
| i. (Did not want to answer) | I |  |
| j. (Don’t know) | J |  |

**9.2 Please CIRCLE the letter that best describes the TOTAL MONTHLY HOUSEHOLD INCOME of all the people in your household before tax and deductions if you know (including grants)**

**HOUSEHOLD INCOME or write down amounts**

| No income | A |  |  |
| --- | --- | --- | --- |
| <R1000 | B |  |  |
| R1001 – R1500 | C |  |  |
| R1 501 – R2 000 | D |  |  |
| R2 001 – R3 000 | E |  |  |
| R3 001 – R5 000 | F |  |  |
| R5 001 – R7 500 | G |  |  |
| R7 501 – R10 000 | H |  |  |
| R10 001 – R15 000 | I |  |  |
| R15 001 – R20 000 | J |  |  |
| R20 001 – R30 00 | K |  |  |
| R30 001 – R50 000 | L |  |  |
| R 50 001 + | M |  |  |
| (Did not want to answer) | N |  |  |
| (Uncertain/Don’t know) | O |  |  |

**THANK YOU FOR YOUR TIME**

**Time__________ RA Sign_____________________**

**_______________________________________________________________________________**

**10. PCR result**

**10.1 POC PCR _________ Time________ Date________________**

10.2 Do you prefer to get your babies result…….

on the same day ☐ at a later date ☐ don’t mind ☐

10.3. How do you feel about your babies result?

**THANK YOU**

**RA Name _________________ Sign_______________ Date____________**
