# Supplementary material for: Point-of-care HIV testing best practice for early infant diagnosis: an implementation study
Source: BMC Public Health. 2019 Jun 11;19:731. doi: 10.1186/s12889-019-6990-z (PMC6560857; doi:10.1186/s12889-019-6990-z)
Supplement: Supplementary file 2 — '10 week' Questionnaire. (DOCX 471 kb) [file 12889_2019_6990_MOESM2_ESM.docx]

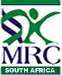

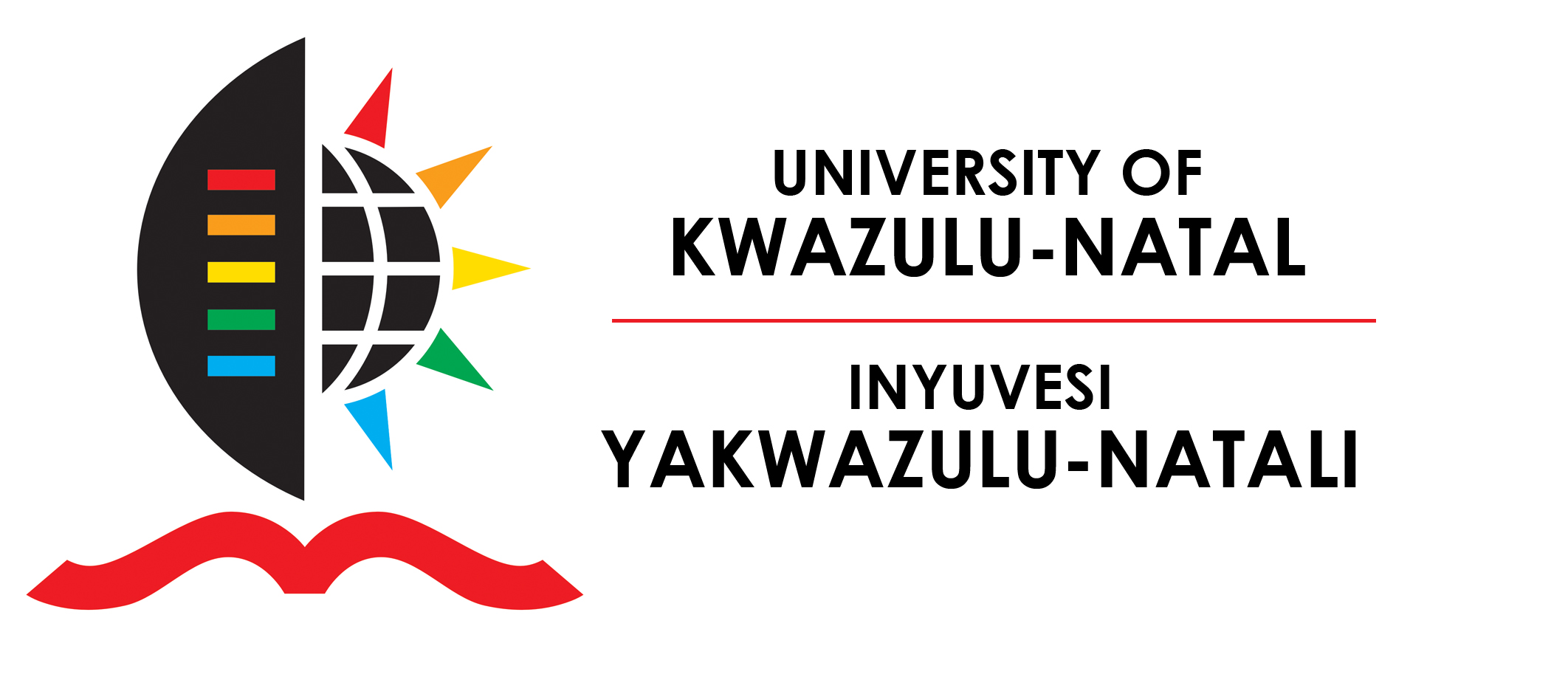


**10week Questionnaire (or later)**

**Study no._______________ Date:________________ Time:____________**

**We would like to ask you some questions to understand how you are getting on with your baby. All the answers you give are confidential and the people reading them will never know whom you are.**

**1. Mother**

1.1 Age☐☐ years 1.2 DOB yy/mm/dd ☐☐/☐☐/☐☐

1.3 Are you a South African Citizen? Yes ☐ No ☐ Nationality _____________________

**2. Caregiver if different to mother**

2.1 Age☐☐ years 2.2 DOB yy/mm/dd ☐☐/☐☐/☐☐

2.3 Care for baby? Full time ☐ Part time(every day) ☐ Part time (not every day) ☐ Occasionally ☐ One off ☐

2.4 Are you… Father ☐ family member ☐ volunteer ☐ paid ☐ ?

**3. Baby - RTHC**

3.1 Gender: M ☐ F ☐ 3.2 DOB dd/mm/yy ☐☐/☐☐/☐☐to confirm

3.3 Birth weight ☐,☐☐kg 3.4 Birth head circumference (HC) ☐☐,☐cm

3.5 Weight today ☐☐,☐☐kg 3.6 Head Circumference (HC) today ☐☐,☐cm

3.7 Feeding: Exclusive Breast ☐ Mixed (breast +formula) ☐ Formula ☐ Solids ☐

3.8 Have you changed the feeding since delivery? Yes ☐No ☐ Date changed_____________

Explain__________________________________________________

____________________________________________________________________________________________________________

3.9 Did you have a POC PCR at Addington?

Yes ☐ No ☐ If yes: Result - Pos ☐ Neg ☐ Unknown ☐

3.10 Did you receive your baby’s birth PCR result from NHLS? (Check RTHC)

Yes ☐ No ☐ If yes: Result - Pos ☐ Neg ☐ Unknown ☐

3.11 **If Positive**: TREATMENT

3.11.1 Did your baby start ARV treatment? Yes ☐ No ☐ Date:_____________

If yes: - If no: go to 3.10.3

3.11.2 Where has your baby been followed up? Hospital ☐ Clinic ☐

Explain: ____________________________________________

________________________________________________________________________________________________

3.11.3 If no, explain:- _____________________________________

________________________________________________________________________________________________________________________________________________

3.12 **If Negative**: PROPHYLAXIS

3.12.1 Was your baby given Nevirapine? Yes ☐ No ☐

3.12.2 Start date?: ______________ 3.10.3 Stop Date?:______________

3.12.4 Was your baby given AZT? Yes ☐ No ☐

3.12.5 Start date?: ______________ 3.12.6 Stop Date?:______________

Other _____________________________________________

3.12.7 Did you run out of Prophylaxis medication (NVP or AZT) from Hospital? Yes ☐ No ☐

If no go to 3.13

If yes, explain _________________________________________

________________________________________________

3.12.8 How many days did your baby miss prophylaxis medication if any? ☐☐days

Explain ____________________________________________

________________________________________________________________________________________________

3.13 Did your baby receive any other PCR / HIV tests? Yes ☐ No ☐ UNK ☐

**4. Birth Registration**

4.1 Has your baby been registered with Home Affairs? Yes ☐ No ☐ UNK ☐

4.2 Done at: Hospital ☐ normal Home Affairs office ☐

Explain _________________________________________________________________________

**5. Immunization**

5.1 Did your baby receive the full immunization at birth? – see RTHC

BCG ☐ Polio ☐ Vit K ☐ If no explain__________________________________

________________________________________________

5.2 Did your baby receive the 6 week immunizations? Yes ☐No ☐ Date________________

Clinic? Lancers Rd ☐ Other clinic’s name ?_______________________________

5.3 If older than 10 weeks are all immunizations up to date? Yes ☐ No ☐

If No …..explain _____________________________________________

________________________________________________________________________________________________

**6. Illness**

6.1 Has your baby been unwell at all? Yes ☐ No ☐ If no go to 7

6.1.1 If yes – Details including treatment received?

________________________________________________________________________________________________

**7. Mothers ART (only if Mother brought child)**

7.1 When did you start ART? ☐☐/☐☐/☐☐☐☐

7.2 Where do you collect your treatment? _______________________________

7.2 What ART regimen are you on? FDC ☐ 2^nd^ Line _________________________________

7.3 When was your last Viral Load test? ☐☐/☐☐/☐☐☐☐?

7.4 VL Result? ____________________________________

**8. Education (Ma or Carer)**

| a. None |  | d. Some Secondary/High school |  |
| --- | --- | --- | --- |
| b. Some Primary |  | e. Secondary/High School completed |  |
| c. Primary completed |  | f. Tertiary/ After school |  |

**9. Partner/ Father of the child (only if Mother)**

9.1 Do you have a partner at the moment? Yes☐ No☐

9.2 Is it the father of the child? Yes☐ No☐

9.3 Do you know the Father’s HIV status? POS ☐ NEG ☐ UNK☐

9.3.1 If positive… on ART? Yes ☐ No ☐ UNK ☐

9.4 Are you married? Yes ☐ No ☐ Divorced ☐ Widowed ☐

9.4.1 if Yes legal ☐ or traditional ☐

**10. Household**

10.1 Could you indicate the type of dwelling that the baby occupies at the moment?

| Dwelling/House or brick structure on a separate stand or yard or on farm | A |
| --- | --- |
| Traditional dwelling/ Hut/ Structure made of traditional material | B |
| Flat or apartment in a block of flats | C |
| Town house /semi-detached house in a complex | D |
| Semi detached house | E |
| House/Flat/room in backyard or in a house | F |
| Informal dwelling/Shack in backyard | G |
| Informal dwelling/Shack not in backyard, e.g. in an informal/squatter settlement or on farm | H |
| Caravan/Tent | I |
| Hostel | J |
| Other, specify ………………………………………… | K |

10.2 How many live in this household? (including you if you stay there)

Adults :- ☐ ☐ Children : ☐☐

10.3 If the Mother - Do you have other children that you gave birth to? Yes☐ No☐

10.3.1 If so, please complete the table?

| ChildNo. | Year born? | Stay with you?  Yes or No | HIV Status?  Pos, Neg, UNK | On ART?  Y, N, UNK |
| --- | --- | --- | --- | --- |
| 1 |  |  |  |  |
| 2 |  |  |  |  |
| 3 |  |  |  |  |
| 4 |  |  |  |  |
| 5 |  |  |  |  |
| 6 |  |  |  |  |

**Food Security**

10.4 In the past [4 weeks/30 days], was there ever no food to eat of any kind in your house because of lack of resources to get food?

Yes ☐ No ☐ (if no skip to 6.7)

10.4a How often did this happen in the past [4 weeks/30 days]? (Circle)

1 = Rarely (1–2 times) 2 = Sometimes (3–10 times) 3 = Often (more than 10 times)

10.5 In the past [4 weeks/30 days], did you or any household member go to sleep at night hungry because there was not enough food?

Yes ☐ No ☐ (if no Skip to 6.8)

10.5a How often did this happen in the past [4 weeks/30 days]? (Circle)

1 = Rarely (1–2 times) 2 = Sometimes (3–10 times) 3 = Often (more than10 times)

10.6 In the past [4 weeks/30 days], did you or any household member go a whole day and night without eating anything at all because there was not enough food?

Yes ☐ No ☐ (if no Skip to 7)

10.6a How often did this happen in the past [4 weeks/30 days]? (Circle)

1 = Rarely (1–2 times) 2 = Sometimes (3–10 times) 3 = Often (more than 10 times)

**11. Employment (if Mother/Father)**

11.1 Which of the following best describes the mothers present work situation?

| a.Employed full time | A |
| --- | --- |
| b.Employed part time | B |
| c.Employed less than part time (casual work/piecework) | C |
| d.Self Employed | D |
| e.Temporarily sick / Maternity leave | E |
| f.Unemployed, not looking for work | F |
| g.Unemployed, looking for work | G |
| h.Student/learner | H |
| i.Permanently sick or disabled | I |
| j.Volunteering | J |
| k.Other (specify) ………………………………………………………………….. | K |

**12. Income**

**12.1 Do you or anyone in your household receive any of the following welfare grants?**

| GRANT |  | Amount? |
| --- | --- | --- |
| a. No-one in household receiving any benefits | A |  |
| b. Old Age Grant / Pension | B |  |
| c. Child Support Grant | C |  |
| d. Disability Grant | D |  |
| e. Care dependency grant | E |  |
| f. Foster care grant | F |  |
| g. Grant in aid | G |  |
| h. Don’t know the name of grant | H |  |
| i. (Did not want to answer) | I |  |
| j. (Don’t know) | J |  |

12.2 Please CIRCLE the letter that best describes the TOTAL MONTHLY HOUSEHOLD INCOME of all the people in your household before tax and deductions if you know (including grants)

**HOUSEHOLD INCOME or write down amounts**

| No income | A |  |  |
| --- | --- | --- | --- |
| <R1000 | B |  |  |
| R1001 – R1500 | C |  |  |
| R1 501 – R2 000 | D |  |  |
| R2 001 – R3 000 | E |  |  |
| R3 001 – R5 000 | F |  |  |
| R5 001 – R7 500 | G |  |  |
| R7 501 – R10 000 | H |  |  |
| R10 001 – R15 000 | I |  |  |
| R15 001 – R20 000 | J |  |  |
| R20 001 – R30 00 | K |  |  |
| R30 001 – R50 000 | L |  |  |
| R 50 001 + | M |  |  |
| (Did not want to answer) | N |  |  |
| (Uncertain/Don’t know) | O |  |  |

**13. Leave**

13.1 Did the babies mother/father need to take leave for today?

Yes ☐ No ☐

13.1.1 If yes, was your leave

|  | YES | NO |
| --- | --- | --- |
| Unpaid leave |  |  |
| Sick leave |  |  |
| Annual leave |  |  |
| Family Responsibility leave |  |  |
| Other…………………. |  |  |

13.2 Can you tell me your loss of earnings coming to the clinic if any?

None ☐ R1-R50☐ R50-R100☐ R100-R150☐ R150-R200 ☐ >R200 ☐

**14. Transport**

14.1 How did you get to the clinic?

Walk ☐ Lift ☐ Own Car ☐ Bus☐ Taxi ☐ Train☐ Other______________

14.2 How long did it take you to get here?

< 15 min ☐ 15-30 min☐ 31-45 min☐ 46-60 min☐

61-90 min☐ 91-120 min☐ >120min ☐

14.3 Total cost for transport coming to the clinic? R____________

14.4 Total cost to get back? R____________

**16. Result**

**POC PCR _________ Time:______________**

16.1 Do you prefer to get your babies result…….?

on the same day ☐ at a later date ☐ don’t mind ☐

16.2 What was your response to your babies result?

**THANK YOU**

**RA Name_______________Sign_________________ Date____________**
