# Supplementary material for: Point-of-care HIV testing best practice for early infant diagnosis: an implementation study
Source: BMC Public Health. 2019 Jun 11;19:731. doi: 10.1186/s12889-019-6990-z (PMC6560857; doi:10.1186/s12889-019-6990-z)
Supplement: Supplementary file 3 — Staff Questionnaire. (DOCX 432 kb) [file 12889_2019_6990_MOESM3_ESM.docx]

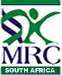

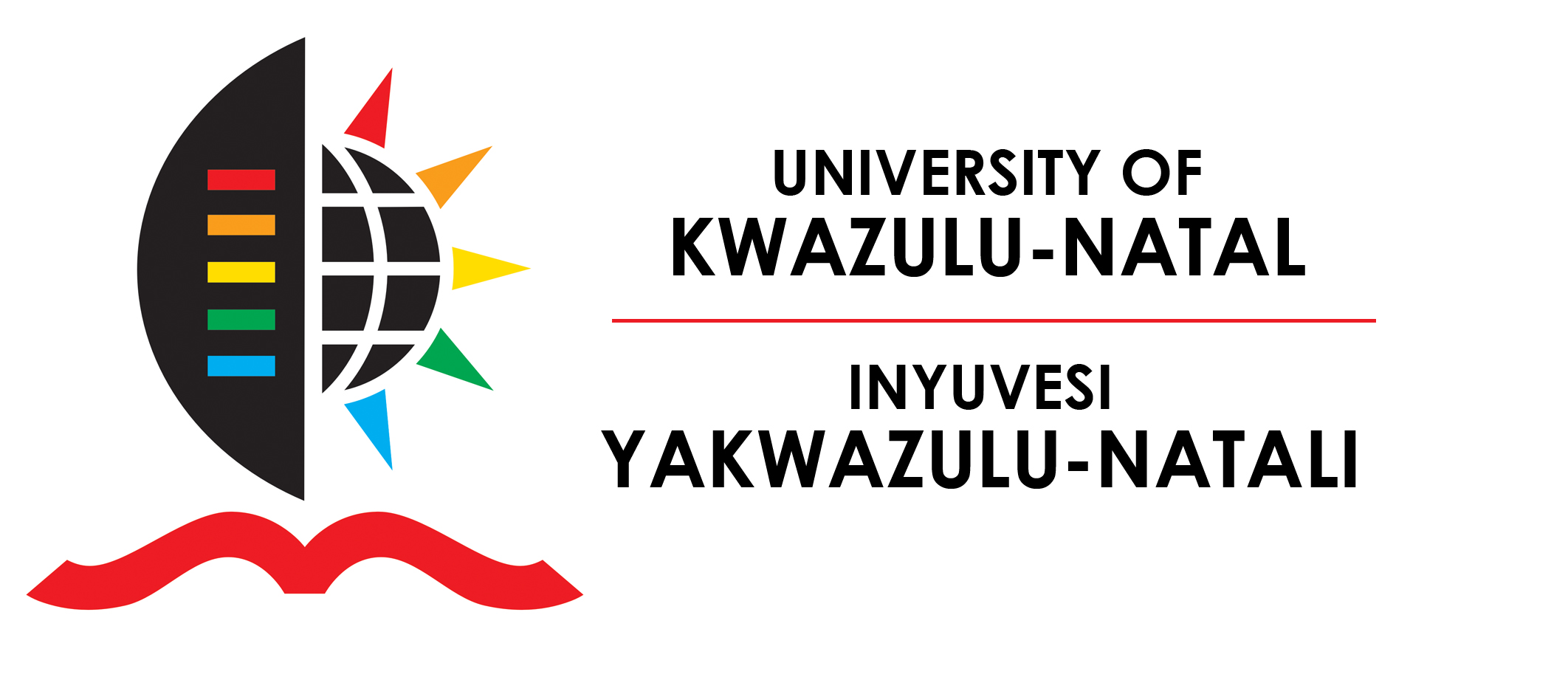


**Staff Questionnaire**

**Staff study no._______________ Date:________________ Time:____________**

**We would like to ask some questions to understand your experience with the Alere Q Detect testing for infant PCRs. All the answers you give are confidential and the people who read this will not know who you are.**

1. How long have you been working in this department?_________________
2. How long have you been taking PCR blood spots from babies? ________________
3. What is your qualification? (circle) RN EN ENA Doctor

Student 1^st^ year Student 2^nd^ year Student 3^rd^ year Other___________________

1. Do you feel adequately trained to perform the PCR blood spot? Yes No if Yes go to 6.
2. If No, explain ____________________________________________________________________________________________________________________________________________________________________________________________________________________________________________________
3. How many blood spots do you usually perform per week on average? _____________
4. Do you have any challenges taking blood spots? Yes No if No go to 8
5. If yes – what are those challenges?

____________________________________________________________________________________________________________________________________________________________________________________________________________________________________________________

1. How many Alere q Detect tests have you done? _______________(can get from register)
2. Have you been adequately trained to do Point of Care PCR testing? Yes No if Yes go to 12.
3. If No, explain

________________________________________________________________________

___________________________________________________________________________________________________________________________________________________________________________

1. Do you have any challenges taking POC PCR tests? Yes No if No go to 14
2. If yes – what are those challenges?

____________________________________________________________________________________________________________________________________________________________________________________________________________________________________________________

1. Of the 2 tests which do you prefer?

Blood spot PCR Detect PCR Don’t mind either

1. Please give reasons if you prefer one or the other

____________________________________________________________________________________________________________________________________________________________________________________________________________________________________

1. Which do you think is better for the Mom and baby?

Blood spot PCR Detect PCR Either

1. If one is better please say why_________________________________________________

___________________________________________________________________________________________________________________________________________________________________________

Thank you for taking time to answer the questions

RA signature_____________________
